# Understanding Neighborhoods’ Impact on Youth Substance Use and Mental Health Outcomes in Paterson, New Jersey: Protocol for a Community-Based Participatory Research Study

**DOI:** 10.2196/29427

**Published:** 2021-05-28

**Authors:** Ijeoma Opara, Noelle R Leonard, Daneele Thorpe, Trace Kershaw

**Affiliations:** 1 School of Public Health Yale University New Haven, CT United States; 2 Silver School of Social Work New York University New York, NY United States; 3 Department of Psychology Stony Brook University Stony Brook, NY United States

**Keywords:** protocol, substance use, mental health, venue-based sampling, community

## Abstract

**Background:**

Substance use among youth is a major public health concern. Of note, substance use among youth is increasing in prevalence, and the incidence of substance use at earlier ages is rising. Given the long-term consequences of early substance use, it is important to identify factors that increase youth vulnerability to drug use, as they may be important targets for future interventions.

**Objective:**

This study aims to use innovative methods, such as venue-based sampling, to recruit youth who are disconnected from school and use community-based participatory research to gain a better understanding of the prevalence of substance use and important correlates among youth aged between 13 and 21 years in Paterson, New Jersey, a low-income, urban community. The study will use a convergent, mixed methods design involving multiple data collection components and the analysis of a ministrative data source, designed with the strengths of complex intervention frameworks in mind. The overall aims of the study are to identify the prevalence of substance use among youth who are engaged in school and not engaged in school; to understand important antecedents and correlates of substance use; and to use this information to inform social, environmental, and culturally appropriate interventions to address substance use and its correlates among youths in a lower-resourced urban community.

**Methods:**

This study will use both qualitative and quantitative methods to address important questions. Specifically, semistructured interviews using focus group and interview methodologies will be used to assess youths’ lived experiences and will account for specific details that quantitative methods may not be able to attain. In addition, quantitative methods will be used to examine direct and multilevel associations between neighborhood factors and youth substance use and mental health outcomes.

**Results:**

A previous analysis from a substance use initiative in Paterson, New Jersey found that youth who use substances such as marijuana and alcohol are more likely to have higher rates of depression and anxiety. On the basis of the research questions, this study will examine the association between neighborhood characteristics, substance use, and mental health symptoms among youth in Paterson by using quantitative and qualitative methods and will use these findings to inform the adaptation of a community- and evidence-based substance use prevention intervention for these youths.

**Conclusions:**

The findings of this study will provide an important contribution to understanding the role of socioecological factors in predicting substance use and mental health outcomes among youth in a lower-resourced, urban community. Furthermore, these findings will serve as evidence for the development of a culturally informed, community-based prevention program to address substance use disparities for youth, including those who are truant in Paterson, New Jersey.

**International Registered Report Identifier (IRRID):**

PRR1-10.2196/29427

## Introduction

### Background

Youth substance use continues to be a major public health issue in the United States, as it has reached epidemic proportions [1-3]. According to the National Institute on Drug Abuse and for the purposes of the current proposal, substance use is defined as the use, misuse, and abuse of substance—both licit and illicit drugs and alcohol. In a national sample assessing drug use among adolescents and teens, approximately two-thirds of students reported they had tried alcohol by the 12th grade, followed by marijuana (45%) and cigarette use (31%) [4]. Furthermore, youth who use substances are at risk of both acute (eg, changes in appetite, wakefulness, heart rate, and blood pressure) and long-term (eg, heart or lung disease, cancer, mental illness, HIV/AIDS, and hepatitis) consequences on their health [5]. Of importance, youth growing up in low-income urban areas are particularly vulnerable to substance use as well as their negative health consequences [6-8]. Given the long-term consequences of early substance use, it is important to identify factors that increase youth vulnerability to drug use, as they may be important targets for future interventions [8]. Urban youth may be exposed to traumatic conditions on a daily basis that affect their mental health, leading them to use poor coping mechanisms such as sexual risk taking and substance use [9,10]. Although interventions exist to tackle this complex issue, these interventions show limited effectiveness and only serve a subsample of youth substance users, given that much of the sampling and dissemination occurs within school contexts. Often neglected in the receipt of these services are youth who are truant or not engaged in school-based services [11-15]. Sampling bias is a consequence of using methods that do not provide all members of the population of interest equal probability of being sampled [13]. This type of error is common in youth substance use surveys, where youth who are engaging in higher levels of substance use are less likely to be sampled and less likely to complete these surveys [14,15]. Nonetheless, the likelihood of falling into a potential sample frame may in some cases be associated with substance use behaviors [13-15]. Therefore, substance use research may be uniquely vulnerable to sample coverage error. Youth who do not attend school on a regular basis or who have dropped out may be more likely to have increased substance use [16,17] and may be particularly vulnerable to the negative effects of substance use. Thus, the systematic exclusion of this population underestimates prevalence estimates of substance use among youth, which can, in turn, affect policy, funding decisions, and the development and generalizability of prevention and intervention efforts. Effective interventions are informed by demographic, contextual, and vulnerability factors as well as the unique experiences and perspectives of individuals in the target population. It is vital for substance use researchers to develop strategies for reducing sampling errors to include a wider representation of youth within a community. Using innovative methods, such as venue-based sampling, to reach the most vulnerable youth, this study aims to understand the effects of neighborhoods on youth substance use and mental health.

### Theoretical Framework

Theoretical frameworks on substance use suggest a range of systemic, environmental, and personal factors that may influence substance use. Consistent with the ecological systems theory of development, the microsystem (ie, most proximal factors including family and peers, such as exposure to substance use or violence at home), exosystem (ie, community, neighborhoods, and school settings, such as community violence and the availability of alcohol supply stores), and macrosystem (ie, most distal factors including policies, cultural beliefs, and values, such as structural disenfranchisement and discrimination) factors as well as their interactions can influence youth substance use behaviors [18,19]. These factors may uniquely affect ethnic minority youth because of greater exposure to microsystem, exosystem, and macrosystem adversities [20]. Indeed, ethnic minority youth in urban communities are more likely to witness drug use, experience traumatic events, and live in underresourced communities, which can all facilitate poor mental health outcomes and drug abuse [21-23]. Furthermore, social disorganization theory posits that individuals are affected by their contexts and that they, in turn, exert influence on those contexts [24,25]. Previous research has documented that particular aspects of social organization (eg, social support and neighborhood closeness) and the presence of community organizations (eg, neighborhood groups and youth-serving organizations) are inversely associated with drug use and poor mental health outcomes [26]. Addressing substance use and mental health among youth in low-resource urban communities can be challenging because of infrastructure, limited resources, and community mistrust [27,28]. To address these barriers, this study will use a social and environmental justice framework to better understand how the intersection of community, culture, and identity can be used to inform interventions to address health disparities in urban communities [29].

One of the fundamental aims of environmental justice research is to investigate if and how environmental resources are distributed inequitably with regard to race and socioeconomic status and its subsequent impact on health. For example, a high density of alcohol outlets creates greater access to alcohol for youths. Unfortunately, in many urban neighborhoods, the high proportion of alcohol outlets is not balanced by other opportunities for recreational development, such as access to parks, playgrounds, afterschool programs, and initiatives for youth success [30]. In addition to resource deprivation, many urban neighborhoods also contend with higher rates of neighborhood threats as a result of increased crime. Indeed, studies have shown that youths who reside in communities with higher crime are more likely to engage in substance use [31] and are more likely to develop mental health problems, such as depression [32], posttraumatic stress disorder [33,34], and anxiety [35]. Thus, using this framework takes into consideration the interaction between the individual and the multiple contexts in which they exist (eg, work, school, and community). Although the environmental justice framework has been applied to address racial and socioeconomic disparities in access to health resources such as recreational opportunities (eg, youth organizations and sporting events) and healthy food access, research on environmental factors that impact substance use and mental health among youth is limited. Historically, much of the literature has focused on individual-level factors that precipitate substance use in youth [36]. Methods are needed to build on the limited environmental justice research to address the environmental risks and resources associated with substance use and mental health by using neighborhood-level variables and community-engaged principles to collect data and develop interventions.

In line with these goals, this study will use a community-based participatory research (CBPR) approach that promotes collaborative and equitable relationships between academic investigators and community partners as well as both culturally and contextually situated intervention development, implementation, and community capacity building processes and outcomes [29]. CBPR recognizes strengths within the community and knowledge of community members and uses youth participants as experts of their lived experiences. Working with both youth in school and youth who are truant to inform prevention interventions is essential to elicit the perspectives of youth with diverse experiences across the entire city. Our study will be guided by our youth and community advisory board, which will consist of 10-12 community partners and 6-10 youth represented in each ward of Paterson.

### Objectives

This study aims to use community-based and innovative methods, such as venue-based sampling and CBPR, to gain a better understanding of the prevalence of substance use and important correlates among youth aged between 13 and 21 years within a lower-income, urban community. Historically, research in this area has focused on individual- and family-level factors that influence youths’ substance use patterns [36]. This protocol will describe the goal of the research; the intended population; and the methods that will be used for sampling, recruitment, and analyses. Specifically, this study will use venue-based sampling and CBPR as a recruitment method to identify vulnerable youth. Specifically, we hope to (1) identify the prevalence of substance use among youth; (2) understand important antecedents and correlates of substance use at the microsystem, exosystem, and macrosystem levels; and (3) use this information to inform social, environmental, and culturally appropriate interventions to address substance use and its correlates among youths in a lower-resourced urban community.

### Study Setting: Paterson, New Jersey

Youth for this study will be recruited from Paterson, New Jersey, a city situated in Passaic County. Paterson is a predominantly low-income, urban, underresourced city in the northeast with a population of approximately 147,000 residents [37]. Paterson is the third largest city in New Jersey, is considered one of the poorest cities, and has one of the highest rates of substance abuse, sexually transmitted infections, and HIV/AIDS in the state [38]. According to the US Census Bureau, approximately 30% of the city’s population lives below the poverty line, with an average household income of US $34,042, which is nearly US $40,000 less than the state’s average income [37]. Paterson’s child poverty rate is 41%, which is higher than New Jersey’s rate of children in poverty (16%). Moreover, 28% of the residents living in this city were aged <18 years. More than 90% of the city’s population identify as either Hispanic (57.7%) or African American or Black (34.7%), and nearly one-third are foreign-born residents [37]. Paterson has a population large and diverse enough to allow for important study comparisons with other urban cities in the nation. Paterson comprises 6 *wards*. Wards are legally defined divisions of neighborhoods for electoral purposes by zip code. The city encompasses a broad spectrum of socioeconomic statuses [37]. Given the disparities that exist within Paterson, it is important to leverage research questions to inform the development of interventions to address the plights of these communities, as these disparities have been shown to increase the risk of substance use.

### Youth Substance Use and Mental Health Trends in Paterson, New Jersey

Local-level data collected by a coalition in Paterson, New Jersey, found that 1 in 4 youth in Paterson used marijuana in the past 30 days [39], indicating that marijuana is the most widely used substance among the youths, followed by alcohol. Compared with other residents in Passaic County, Paterson residents have higher access to alcohol because of the high number of alcohol outlets in the city [39]. Thus, Paterson is a unique city to investigate and uses venue-based sampling techniques for this study because of the following reasons: (1) significant representation of the 2 largest ethnic groups in the nation (Black and Hispanic) that reside in Paterson and (2) the city’s wide availability of licit and illicit drugs that may impact community norms and values. For example, based on the parameters set by the New Jersey Division of Alcoholic Beverage Control [40], the city of Paterson with more than 147,000 residents should not exceed a total of 49 consumption licenses, including restaurants and bars, and 19 off-premises licenses, including liquor stores and bodegas. However, Paterson is only 8.4 square miles and has more than 200 alcohol outlets—4 times the legal limit because of grandfather clauses [37]. Alcohol outlet density is not only associated with increased crime, violence, and heavy alcohol and drug use but also provides Paterson youth with greater access to alcohol, leading to an increased probability of underage drinking [41-43]. Among Paterson youth aged between 13 and 18 years, 30% of youth reported that they had used alcohol before the age of 13 years [39]. Local-level data show that Paterson youth who drank alcohol during the past 30 days were also 3 times more likely to smoke marijuana before the age of 14 years [39]. Of note, 60% of Paterson youth reported purchasing alcohol from liquor stores and 40% of Paterson youth admitted to having an adult purchase alcohol from liquor stores for them [39]. Furthermore, Black (33.3%) and Hispanic (29.7%) Paterson youth reported the highest marijuana use before the age of 13 years [39]. In addition, the city is currently facing an extreme opioid crisis, with Paterson being ranked the second city in New Jersey for the highest rate of heroin abuse [44]. Among Paterson youth, 6.2% reported using cocaine and 6.8% reported using heroin; these rates are higher than the state averages [39]. These findings highlight the need to address substance use among Paterson youth, specifically, investigating antecedents and correlates to inform interventions to reduce substance use among youth. Although empirical studies suggest a bidirectional relationship between substance use and mental health [41], it is important to acknowledge that they are correlated and may influence each other, leading to poorer short- and long-term outcomes. Indeed, among Paterson youth sampled, there is a high mental health burden, as 50% of youth reported experiencing depressive symptoms and 57% reported experiencing anxiety symptoms [39]. Overall, 70% of youth who used either alcohol or marijuana in the past 30 days reported experiencing greater symptoms of anxiety and depression [39]. Given that both depression or anxiety symptoms and substance use are correlated and are associated with other negative health outcomes [45,46], it is important to understand these associations to inform and improve interventions aimed at decreasing mental health disparities and limiting substance use.

## Methods

### Overview

The CHERRIES (Checklist for Reporting Results of Internet E-Surveys) checklist recommendations for authors presented by the Journal of Medical Internet Research [47,48] are provided in Multimedia Appendix 1 in an effort to ensure complete descriptions of web-based surveys.

### Participant Recruitment and Sample Size

The eligibility criteria for study participation included youth who were (1) aged between 13 and 21 years, (2) able to speak and read in English, and (3) reside in Paterson, New Jersey. We acknowledge that Paterson is a very diverse city with residents who speak several languages besides English. The study intends to take into account differences that will arise in age, race, socioeconomic status, and gender and will work with the community to develop appropriate interventions informed by findings to be most effective in preventing substance use and improving mental health outcomes of youth [49].

The study will use a convergent [50], mixed methods design involving multiple data collection components and the analysis of a ministrative data source, designed with the strengths of complex interventions framework in mind. Venue-based sampling [51,52], purposive sampling [53,54], and snowball sampling [55] will be the 2 primary sources of recruitment for participants. The youth and community advisory board will be used as key informants to identify places where youth often congregate when they are not in school. Studies that focus on youth and use venue-based sampling have demonstrated that they can tap into at-risk groups and provide important information about environments and hard-to-reach populations to better inform interventions. Purposive sampling will involve recruiting youth through partnering youth-serving organizations and schools that have direct access to youth in the specified age range by using venue-based sampling.

Purposive sampling [53,54], also referred to as *judgmental* or *expert sampling*, is a type of nonprobability sample. The main objective of a purposive sample is to produce a sample that can be logically assumed to be representative of the population. This is often accomplished by applying expert knowledge of the population to select a sample of elements that represents a cross-section of the population in a nonrandom manner. The youth and community advisory board will assist in recruiting eligible participants by passing out flyers and emailing listservs.

Snowball sampling [55] is a recruitment technique in which research participants are asked to assist researchers in identifying other potential subjects. To justify the use of this technique, because we are seeking to collect data from participants who ideally would be *difficult* to reach through conventional and traditional methods of recruiting youth, snowball sampling is a useful technique for working with marginalized and hard-to-reach populations.

Venue-based sampling [51,52] is a recruitment strategy that entails targeted recruitment through preidentified venues in Paterson, New Jersey, where youth congregate. This sampling method will occur in 1 of 2 ways. The youth and community advisory board will be used as key informants to identify places where youth often congregate when they are not in school. We will outline 10-20 venues, and then the research team will visit those venues and attempt to recruit youth to participate in the brief survey. Youth will also have the opportunity to be a part of the qualitative part of the study at another time. In addition to venue-based sampling, participants will also be recruited through various sources, such as community partner organizations, through social media channels (Facebook, Twitter, and Instagram) and purposive and snowball sampling.

### COVID-19 Contingency Plan

Pending COVID-19 restrictions, we will disseminate the survey on the web and ask youth to identify the venues where they have been in the past 7 days and popular venues where they tend to congregate. After the first wave of participants have identified venues, the research team will visit venues and attempt to recruit additional participants while also observing the surroundings of the selected venues to determine characteristics that can be seen as risky or protective (eg, needles on the ground, number of liquor stores close to the venue, parks, and libraries).

### Procedures

#### Overview

Demographic variables such as race or ethnicity, sexual orientation, religiosity, and socioeconomic status will be collected. The study will use both qualitative and quantitative methods to obtain a more comprehensive understanding of the antecedents, correlates, and consequences of youth substance use. With regard to recruitment, we will seek a waiver to consent to youth without parental consent for several reasons: (1) to protect the youth participants’ privacy regarding their involvement in using drugs or substances, mental health treatment, and experiencing mental health symptoms; (2) to ensure youth receive mental health treatment (if needed); (3) to encourage participants to give honest answers to the study questions (without fear of parent or guardian reactions); and (4) to be in line with state and federal guidance on the issue. Previous research on youth suggests that requiring parental permission may decrease their interest in participating in biobehavioral research [56,57]. Requiring parental permission would also violate local and federal rights and breach confidentiality for youth whose parents or guardians do not know that they were receiving mental health treatment, engaging in substance use, and possibly seeking mental health services [57-59]. Youth who are interested in participating will be provided with a detailed consent form outlining their participation in the study. Research staff will be available to answer youths’ questions about the consent form and their participation. After the youth complete either the survey or the focus groups, they will be debriefed about their participation in the study. Given that some of the information asked may be sensitive to youth, we expect minimal risk. The research team, however, will provide youth with resources to access services (ie, mental health and substance use treatment). Youth are able to discontinue the survey at any time without penalty or request that their data be redacted. Youth will be compensated US $10 for their participation in the survey portion of the study.

#### Qualitative Interview Outcomes

Qualitative methodology allows participants to discuss their lived experiences and can account for specific details that quantitative methods may not be able to attain. The research team will conduct semistructured interviews using the focus group methodology. In addition, a subsample of these participants will conduct individual interviews with a member of the research team. Research questions will address the factors that contribute to substance use in Paterson. Semistructured individual interviews will be conducted after focus groups to clarify themes and explore deep issues in greater depth. The target sample size for the focus groups will be 100 youths. A subsample of youths (N=50) will be asked to participate in individual interviews following the focus group interviews. The interview guide will consist of questions pertaining to substance use perception and knowledge, mental health status, and needed neighborhood resources. This component of the study focuses on 3 specific research questions: (1) what are the social and environmental contexts of substance use initiation; (2) how do youths define mental health symptoms such as anxiety and depressive symptoms; and (3) what resources do youth in Paterson identify to be beneficial in reducing or preventing substance use?

#### Qualitative Data Collection

First, participants will be asked to complete a 60- to 90-minute focus group with a trained facilitator. Focus group methodology provides insight and understanding of the phenomena by allowing the researcher to examine interactions among participants [60]. Focus groups will be stratified by age (eg, 12-13, 14-15, 16-18, and 19-21 years), gender (eg, male, female, and transgender), and race (eg, Black, Hispanic, and Bangladeshi). We hypothesize significant differences in risk and protective factors for substance abuse and mental health by group. Each focus group will comprise 5-8 youth participants. Although previous research indicated that 3 focus groups can yield saturation [61], we anticipate conducting 12 focus groups to improve study rigor and account for saturation within each subgroup of youth. For each focus group, 2 facilitators will be involved. Facilitators will be closely matched to youth participants by race, ethnicity, and gender. Following the focus groups, a subsample of (N=50) youth will be asked to participate in individual interviews to triangulate the data and delve deeper into sensitive themes that arose in focus groups. The lead facilitator will also interview the participants individually. The second cofacilitator will take notes of observations of nonverbal exchanges, including displays of emotion and nervous gestures that took place during the interviews. Adaptive questions will be used to further understand the unique experiences that youths are sharing. All interviews will be recorded and transcribed. As the interview process develops, questions may be tailored based on the youths’ feedback. The youth and community advisory board will review, revise, and approve the focus group and individual interview questions. Participants will receive US $25 at the end of the focus group interview and US $25 at the end of individual interviews.

#### Quantitative Data Collection

For each venue identified, the informants (members from the youth and community advisory board) will provide the name, address, type of venue, and preferred time. Youth and Community Advisory Board members and research staff will visit the venues identified. Parental consent will be requested to be waived because of confidentiality and sensitivity of the study for youth aged <18 years [60]. On agreeing to the study, participants will be asked to complete a brief 15- to 20-minute open survey onsite (eg, using an iPad or paper format version). Participants will also have the opportunity to schedule to complete the survey at a community-based partnering organization and schedule to be a part of the focus group. We will recruit at least 720 youths through venue-based sampling at venues and purposive sampling through community partners (eg, schools and youth-serving organizations) to complete surveys within the first 2 years of the project. The youth will be provided with the survey via a web-based platform. Youth will provide their responses to the survey questions in a secure location. They will be honest with their responses and will have the ability to change or alter previous responses. Surveys will be administered via Qualtrics, a secure survey tool, through the principal investigator’s university license. Questions in Qualtrics will be presented in a randomized format for each participant. Responses will be automatically populated in Qualtrics. The principal investigator will have sole access to the Qualtrics account and will download all the data once we have reached the intended number of participants for the study. Deidentified response data will be stored using a unique code representing each participant. Data will be stored confidentially via the secure databases of principal investigators’ institutions (eg, SPSS [IBM Corporation]). The attribution rate in the study will be operationalized as the number of participants who complete the consent to participate but did not complete the full study. Complete data will be used for the research analysis.

### Measures

#### Tobacco, Drug, and Alcohol Use

We will measure past 30-day drug use and lifetime drug use using items from the Centers for Disease Control and Prevention Youth Risk Behavior Survey [62]. The following drugs will be measured: alcohol, marijuana, tobacco, e-cigarettes or vape pens, hookah, opioids, and marijuana. An example of an item is “How many times did you use marijuana or hashish in the last 30 days?” Participants respond to these items on a 7-point scale (1=0 days; 2=1-2 days; 3=3-5 days; 4=6-9 days; 5=10-19 days; 6=20-29 days; and 7=all 30 days).

#### Depressive and Anxiety Symptoms

The Brief Symptom Inventory [63] will be used to measure the symptoms of anxiety and depression. Six items assess the frequency with which participants felt uncomfortable during the past week because of anxiety symptoms such as “nervousness or shakiness inside” and “feeling tense or keyed up.” An additional 6 items assess the frequency with which participants had felt uncomfortable during the past week because of depressive symptoms such as “feeling blue (or sad)”and “feelings of worthlessness.” Response options range from 1 (not at all) to 5 (extremely).

#### Neighborhood Drug Availability

Neighborhood drug availability [64] will be measured using an adolescent report of 3 items. Adolescents indicate their agreement with each statement on a scale ranging from 1 (strongly disagree) to 5 (strongly agree). Example items include “Marijuana would be easy to find in my neighborhood,” “I know where to get drugs in my neighborhood,” and “Lots of drugs are sold in my neighborhood.” A mean score will be computed such that high scores indicate greater neighborhood drug availability.

#### Neighborhood Resources

Neighborhood physical resources, such as libraries and parks, have been found to contribute to healthy development [64] and play a role in shaping observations and interactions with other residents. Four items asking about the availability or presence of (1) sidewalks or walking paths; (2) parks of playgrounds; (3) recreation, community center, or clubs; and (4) library or bookmobile will be used as indicators of the latent construct of neighborhood resources. Questions will be answered as no (0) or yes (1).

#### Neighborhood Safety

Two questions that ask about (1) feelings of safety in the community or neighborhood and (2) feeling safe at school will be used to indicate this latent construct [65]. These questions will be answered on a 4-point scale, from 1 (never) to 4 (always). Higher scores indicate feelings of safety.

#### Neighborhood Poverty

We will define neighborhood as a geographic unit and will measure it based on the census tract of residence. Census tracts commonly serve as proxies for neighborhoods and are often the basis for geographically delimited resource allocation. Census tract–level data will be acquired on the percentage of residents within each census tract that is at or below the poverty line. Furthermore, mean household income across the census tract will also be gathered as a continuous indicator of neighborhood-level income.

#### Everyday Discrimination

The Expanded Everyday Discrimination Scale [66] is a 10-item measure that will be used to assess the levels of everyday, chronic discrimination. Youth will be asked to identify what they believe to be the main reason or reasons for their experiences (eg, gender, race, and age). Youth will be asked to report on how often any of the following things happen to them (eg, “you are treated with less courtesy than other people are”) on a 5-point Likert scale (1=never and 6=almost every day). Scores on the Expanded Everyday Discrimination Scale range from 10-60, with higher scores indicating higher levels of chronic discrimination.

#### Extracurricular Activity

Youth will be asked to report all of their extracurricular activities from the past year. Specifically, youth will be provided with a list of activities and will be asked to indicate their involvement in these activities. Extracurricular activities were grouped into the following 5 categories based on previous research: school involvement or activities—school band, drama, and dance; team sports; and academic clubs—and community involvement or activities—including church attendance and volunteer and community service type activities. In addition to indicating activities they are involved in, youth will also be asked follow-up questions about each activity [67]. Specifically, they will be asked about (1) the frequency of their participation or involvement ("one day", "two or three days", "four or five days", or "six or seven days"), (2) their enjoyment of these activities ("never", "sometimes", "usually", or "always"), and (3) their length of involvement ("less than one year", "one year", "two or three years", "four or five years", or "six years or more").

## Results

### Overview

A previous analysis from a community-based substance use initiative in Paterson, New Jersey, that the principal investigator was a part of, found that among youth living in the first ward of Paterson (eg, Paterson consists of 6 wards), 1 in 4 youth admitted to using marijuana in the past 30 days [39]. Marijuana is thus the most accessible substance to Paterson youth, followed by alcohol. Paterson residents have extreme access to substances including alcohol because of the high number of alcohol outlets in the city. The factors that make Paterson an important city to study for this project are (1) the significant representation of the 2 largest ethnic groups in the nation (Black and Hispanic) that reside in Paterson and (2) the city’s wide availability of licit and illicit drugs that may impact community norms and values. For example, based on the parameters set by the New Jersey Division of Alcoholic Beverage Control [40], the city of Paterson consists of more than 147,000 residents and should not exceed a total of 49 consumption licenses (restaurants and bars) and 19 off-premises licenses (liquor stores and bodegas). However, Paterson is only 8.4 square miles and has more than 200 alcohol outlets—4 times the legal limit [39]. Alcohol outlet density is not only associated with crime, violence, and heavy alcohol and drug use but also provides the youth in Paterson access to alcohol, leading to underage drinking [41-43]. Among Paterson youth within the first ward, between the ages of 13 and 18 years, 30% of Paterson youth have used alcohol before the age of 13 years [39]. Local-level data show that Paterson youth in the first ward, who drank alcohol during the past 30 days, were also 3 times more likely to smoke marijuana before the age of 14 years [39]. Given the emerging evidence that has been disseminated from this community, it is essential to develop more robust procedures to understand the impact of the environment and neighborhoods on drug use and mental health among Paterson youth.

The study is currently in the first phase of planning and will begin recruitment at the end of year 1 (August 2021) and will complete recruitment at the end of year 3 or when the target number has been reached. By year 4, the pilot intervention will be introduced to the community, and participants will be enrolled to participate. The study is currently under review at the principal investigator’s home institution (Yale University). The study was funded as of September 2020 by the National Institutes of Health Office of the Director. Funding will be completed by August 2025. The results of the study will be published on an ongoing basis.

### Data Analysis Plan

#### Quantitative Data Analysis

We will first discuss common issues that will guide data analysis and then describe the analytical methods specific to the proposed research. We will check for assumption violations by assessing normality, distribution, and collinearity issues. Appropriate variable transformations will be applied to severely nonnormal variables [68]. Multicollinearity will be tested by calculating variance inflation factor and tolerance. We foresee that some data will be missing at random. We will examine patterns and missing variables (ie, completely at random, missing at random, or missing not at random) [68]. Structural equation modeling (SEM) with full information maximum likelihood can account for missing at random [69]. We will perform a sensitivity analysis if there are data missing at random.

#### Outliers

We will be sensitive to outliers in all analyses. Potential problems with outliers will become evident as we compare the results of conventional and robust analyses. We will apply standard methods for outlier detection (eg, analysis of leverage statistics and residuals) and use graphical approaches as well.

#### Statistical Power

In general, statistical power should be adequate. An SEM model with a degree of freedom as low as 20 and a sample size of 300 will have 96% power to build a good-fit model (ie, root mean square error<0.05) [69,70]. With the target sample size (N=720), the study will easily have more df and will achieve higher power to build and test the proposed SEM models. For a multiple regression equation with 5 predictors where the squared multiple correlation is 0.25 and where one wants to detect a predictor that accounts for at least 5% unique variance in the outcome, the required sample size to achieve a power of 0.80 is approximately 115 [68]. As another example, for a logistic regression analysis in which the target predictor is a continuous predictor with 5 other predictors in the equation where the event rate at the mean of all predictors is 0.40 and where the multiple correlation of the predictor with the other predictors is 0.30, the sample size needed to detect an odds ratio (applied to standardized metrics) of 1.75 is 115 [68].

#### Statistical Tests

Owing to the nature of the aims and the hypothesized outcomes, a more advanced and robust test of statistical relationships is needed for this study. SEM is a multivariate statistical analysis technique used to analyze structural relationships using a conceptual model, path diagram, and system of linked regression-style equations. SEM is the combination of factor analysis and multiple regression analysis and is used to capture complex and dynamic relationships between observed and unobserved variables [69,70]. SEM approaches are well-suited for this study because of their ability to deal with latent variables and the assessment of complex mediating relationships in causal analysis. On the basis of hypothesized model mentioned earlier, we predict that neighborhood characteristics will have a direct effect on youth substance use and depressive and anxiety symptoms. These effects will account for youth age (and possibly gender, race, and socioeconomic status). SEM is considered a confirmatory technique, as it extends the possibility of relationships among latent variables and includes 2 components: (1) a measurement model (eg, confirmatory factor analysis) and (2) a structural model. SEM allows the following: (1) more accurate estimates of the effects of hypothesized causal variables, (2) it allows the researcher to estimate all effects simultaneously, (3) greater accuracy of parameter estimates when examining competing models, and (4) it allows the researcher to compare the effects of multiple mediators. To use SEM, there are several assumptions that must be met: (1) a linear relationship is present between endogenous and exogenous variables; (2) there should be a cause-and-effect relationship between the endogenous and exogenous variables that are being tested based on theory; (3) data should be free of outliers because outliers can affect the significance of the model; and (4) there should be a nonspurious relationship between the endogenous and exogenous variables. Nonspurious relationships assume that the relationship between 2 variables cannot be explained without a third variable; (5) equations between variables must be greater than the estimated parameters; (6) a sample of at least 150-200 is preferred with at least 10-15 indicators; (7) error terms among endogenous variables and exogenous variables are assumed to be uncorrelated with other variable error terms; and (8) interval data are to be used for analysis [69,70].

#### Qualitative Data Analysis

We will use 2 different methods of qualitative data collection: focus groups and semistructured individual interviews. The combination of focus groups and individual interviews will make 3 unique contributions: (1) provide an iterative process whereby an initial model of the phenomenon is guided by the description and exploration of individual accounts; (2) identify sensitive cultural, environmental, and contextual circumstances through individual interviews, adding interpretation of the phenomenon; and (3) allow for central and common characteristics to be further explored across focus groups with members who share similarities [68]. In qualitative research, data collection and analysis occur simultaneously. Analysis of interview data proceeds inductively through the identification of recurring themes and patterns in transcripts, field notes, and analytic memos. A thematic analysis framework will be used in this study. The thematic analysis allows researchers to highlight the similarities and differences across groups of participants [61]. The research staff will work from an essentialist or realist perspective that assumes that the participant’s language reflects their experiences, meanings, and realities. Meaningful analytical units will then be developed using a coding scheme informed by the dominant themes in the data. Topics will then be divided into several subtopics based on recurring themes within the larger topics, allowing a more in-depth analysis and complex understanding and interpretation of each particular theme. Each theme and subtheme will be assigned a code, and the codes will be compiled in a codebook. All interviews will be conducted by the research staff. As analysis is taking place, questions for individual interviews will be developed as focus group data are being analyzed. Individual interview data will be analyzed using the same steps used for the focus group data. We hypothesize significant differences in the experiences and perceptions of youth in the study based on their age, race, gender, and socioeconomic status.

#### Strategies for Trustworthiness

Members of the research team will have prolonged engagement within the community to gain a better understanding of the organization in which participants were recruited from and to establish trust with participants. A confirmability audit will be conducted, where multiple coders will be used to analyze the data. The process will be the same for both the individual interviews and focus groups. Data from the interviews will first be analyzed by interviewers using open coding, whereby concepts are identified and labeled as they emerge from the data and across the interviews. Interviews will be transcribed and analyzed using NVivo 12 (QSR International) software [71]. The coding process will be inductive in nature and consist of categorization and grouping. Line-by-line coding will be used, and common themes will be grouped together using a coding map created from NVivo to conceptualize the themes until 4 main categories are identified. At least 90% interrater reliability will be achieved before the codes and categories are developed. The categories that will be developed from the coding process will not be predetermined but rather formed during the coding process. After the initial coding of the data, the research team will summarize and organize the results in NVivo [71].

#### Integration

The analysis of quantitative and qualitative data will initially be conducted separately. Subsequently, we will merge all sets of findings. Member checking of the data will occur in townhalls and disseminating findings throughout the community in the form of infographics and educational materials. The aim of this approach is to balance the respective strengths and weaknesses of each method to maximize the yield of distinct complementary sources of evidence. The main statistical software package that will be used for this project to analyze data and to evaluate the project as well as analyze survey data will be SAS version 9.4 [72], and NVivo will be used to analyze qualitative findings [71].

## Discussion

### Principal Findings

Researchers have struggled with ways to achieve community-based samples that are representative and include an adequate number of youths. In addition, understanding what prevention methods work with youth who are most in need of substance abuse and mental health prevention services, such as street-involved and truant youth, is crucial, and their input is essential, yet their voices are often ignored because of the challenges in reaching this population. Such an absence may make it difficult for interventions to be truly effective for this population. This study proposes innovative methods such as venue-based sampling to recruit and engage with a hard-to-reach population in Paterson, New Jersey. Few studies have used venue-based sampling in youth studies; however, those that have, offered beneficial results through recruitment of hard-to-reach youth [52,73]. A majority of youths are recruited from schools; however, this can lead to bias and neglect of youth who are truant or not connected to youth-serving organizations. Venue-based sampling has the advantage of allowing a comprehensive focus on a single, entire community that can be translated into an effective, culturally tailored, community-based intervention. Venue-based sampling also allows research teams to be fully immersed in the community as an outreach. Through this method, we intend to recruit participants to be a part of our study for data collection and also simultaneously connect youth to our youth-serving community partners. Through this approach, youth become connected to organizations that can serve their needs while contributing their knowledge and experiences as experts of their lived realities to prevention science.

In the final phase of the study, we will use the data to design and iteratively adapt an evidence-based substance abuse and mental health prevention intervention accounting for gender, race, and developmental differences. As we anticipate significant differences across subgroups of Paterson youth, the adaptation of multiple interventions will be expected to fit their diverse needs and experiences. The process of modifying an evidence-based intervention without contradicting its core elements or internal logic is referred to as *adaptation*. Without attention to the cultural context of a new target population, adapted interventions may remain faithful to the underlying theoretical framework and core elements on which they were originally developed but, unfortunately, may lack relevance, sustainability, and acceptability for the target population [29]. Therefore, to effectively implement a successful evidence-based intervention, this study will use assessment, decision, administration, production, topical experts, integration, training, and testing (ADAPT-ITT) [74], a well-known implementation science framework. The framework specifies 8 steps for adapting interventions to new priorities. The first step involves the assessment of service users’ needs, which guides decision making about the modifications that are needed (step 2). The third step involves intervention adaptation and initial administration of the program to potential service users to identify where the intervention requires revision to improve its relevance and acceptability. Thereafter, intervention production occurs in which further adaptations are made (step 4), and this revised intervention is reviewed by topical experts (step 5). Steps 6 to 8 involve integrating all feedback into a final version of the intervention, training staff to deliver the intervention, and feasibility testing of the intervention. The youth and community advisory board and research team will be involved in the adaptation of evidence-based interventions for Paterson youth. As we hypothesize that significant differences will arise in risk and protective factors for youth throughout Paterson, we anticipate adapting a session-based prevention intervention that is structured, informative, and engaging in youths of all ages. Although there are a number of community-based prevention interventions that are geared toward substance use and mental health among urban youth, pending initial data collection and community input, we anticipate adapting the CASASTART (Center on Addiction and Substance Abuse Striving Together to Achieve Rewarding Tomorrows) [75] and Project Towards No Drug Abuse [76].

CASASTART [75] has been identified as a model substance abuse prevention program. CASASTART was originally developed as a substance abuse and violence prevention program serving high-risk adolescents and their families living in socially distressed neighborhoods [75]. The program is a comprehensive, neighborhood-based, school-centered model that aims to provide coordination among police, schools, and community-based organizations to achieve 3 goals: (1) to redirect and build resiliency in the lives of youth who are at risk of using drugs, becoming delinquent, or dropping out of school; (2) to reduce and control illegal drug use and related crime in the neighborhoods where youth live to make the areas safer and the environment more nurturing; and (3) to connect youth and their families to services critical to their well-being in the environment [75]. Another community-based prevention intervention for youth is the Project Towards No Drug Abuse [76] program, which is a 12-session curriculum for youth that targets substance use and violence-related behaviors through the use of a motivation, skills, and decision-making approach. Using interactive teaching techniques, Project Towards No Drug Abuse provides cognitive motivation enhancement activities, information about the consequences of drug use, correction of cognitive misperceptions, communication and coping skills enhancement, and tobacco cessation techniques to students. Although both of these model interventions have shown efficacy in improving knowledge, reducing risky behaviors, and improving mental health outcomes; these interventions often do not reach the most vulnerable youth who are in need of such services as such interventions are performed in school settings [12]. Reaching the most vulnerable youth who may not be connected to schools or organizations or are street-involved is key to effectively addressing youth substance use and mental health as their input on barriers to prevention and treatment are vital to intervention development. Given the knowledge that researchers have on prevention interventions for youth, community-based and culturally relevant interventions are the gold standard for urban communities [77]. It is essential that researchers aim to work with communities on developing solutions that are innovative, specific to their needs, incorporate community members, and reach a wide array of youth who may have various risk factors that need to be addressed in prevention intervention initiatives.

According to the prevention principles proposed by the National Institute on Drug Abuse [78], “Community prevention programs that combine two or more effective programs can be more effective than a single program alone.” Building on these guiding principles, we aim to adopt a comprehensive substance abuse prevention approach that includes mental health education and resource connecting through intensive case management, community mobilization, and interactive education-based interventions. On the basis of prior research, this multitiered strategy has proven to be an effective method for reducing substance abuse risk among urban minority youth [78] CASASTART [75], uses a case management approach in which an individual (trained case manager) works with up to 15 children and their families and directly provides or, through appropriate referral, coordinates a comprehensive menu of services for the youth and their families. The model recommends that each community should implement the intervention and develop its own approach to design and deliver services consistent with local culture and practice. Within this model and with youth and community advisory board approval, we will have a research assistant trained in social work or counseling, provide services that specifically include referring youth to nearby mental health clinics, mentoring programs, and extracurricular activities to keep youth engaged. The case manager will perform a biweekly follow-up with participants to ensure that they are supported and engaged in recommended activities.

The Project Towards No Drug Abuse [76] is a 12-session curriculum that targets substance use and violence-related behaviors through the use of motivation, skills, and decision-making approaches. The curriculum comprises 12 sessions, approximately 45 minutes each, which are designed to be implemented over a 4-week period. Using interactive teaching techniques, the instruction to participants provides cognitive motivation enhancement activities, information about the consequences of drug use, correction of cognitive misperceptions, communication and coping skills enhancement, and tobacco cessation techniques. Although this intervention was originally intended to be implemented in alternative schools, it also provides flexibility to be adapted for youth who are not in school [76]. We have the commitment of community partners with large access to youth both in school and outside of school, which will provide us with space within the community to pilot-test the intervention.

The last phase of the project includes a pilot feasibility trial of the adapted intervention among a sample of youth living in Paterson, New Jersey. During monthly meetings with youth and community advisory board members, research staff will discuss the use of evidence-based prevention interventions that address youth substance use and mental health. A specific strategy and an evidence-based intervention (eg, Substance Abuse and Mental Health Services Administration’s National Registry of Evidence-based Programs and Practices) will be decided through findings collected from venue-based sampling methods and suggestions from the youth and community advisory board. Participants will be recruited from partnering youth organizations to participate in the intervention. We will pilot-test the intervention with a group of youths who are similar developmentally by age, race, ethnicity, and gender. Youth will be sampled from schools and youth-serving organizations across all 6 wards. Although interventions have the power to increase perception, increase knowledge about substances, and reduce risky behaviors, we understand that youth are nested in their environments. Therefore, we are cautious of being ambitious in our goals, as changes cannot occur if the community norms remain the same. Consequently, we will meet the youth and community advisory board to decipher the most effective mode of intervention that can occur at the individual and community levels. We intend to pilot a combination of effective strategies (eg, prevention education, case management, and referral to therapy). We will first identify and adapt an evidence-based intervention that will incorporate elements of mental health discussions, substance use, and refusal strategies, as these have proven to be the most effective among ethnic minority youth. In addition, and most importantly, the intervention will be informed by findings from participants and youth and community advisory board discussions. Although we discussed earlier the flaws in primarily recruiting and working with youth in schools only, the priority will be to adapt an existing prevention intervention to be feasible for youth who are in school and other youth who are not engaged in school. We hypothesize that unique developmental, cultural, racial, and gender-specific differences will arise and inform how to adequately decide, adapt, and deliver an effective prevention intervention for Paterson youth.

### Conclusions

This study will have a high impact on prevention research for the following reasons: (1) it targets youth who may be disengaged from services and schools and thus neglected in prevention research; (2) it incorporates an environmental context-sensitive approach to understanding substance use and mental health outcomes; (3) delineates the pathways linking specific contextual factors with the behavioral determinants described in a well-tested theoretical model of social disorganization and socioecological theories; and (4) creates a strong youth and community advisory board with mutual ties among local stakeholders, educators, mental health clinicians, and youth who will be essential to conduct ethical research and to translate the research into a sustainable prevention intervention for youth in Paterson, New Jersey, and urban cities alike. Study findings will have an impact on how substance use and mental health researchers understand how to work with youth and develop programs specifically targeted for them to ensure sustainability.

## Appendix

Multimedia Appendix 1 CHERRIES (Checklist for Reporting Results of Internet E-Surveys).

## Abbreviations

ADAPT-ITT: assessment, decision, administration, production, topical experts, integration, training, and testing
CASASTART: Center on Addiction and Substance Abuse Striving Together to Achieve Rewarding Tomorrows
CBPR: community-based participatory research
CHERRIES: Checklist for Reporting Results of Internet E-Surveys
SEM: structural equation modeling

## References

[1] Feinstein EC, Richter L, Foster SE (2012). Addressing the critical health problem of adolescent substance use through health care, research, and public policy. J Adolesc Health.

[2] Kaminer Y, Winters KC (2011). Clinical Manual of Adolescent Substance Abuse Treatment.

[3] Conway KP, Vullo GC, Nichter B, Wang J, Compton WM, Iannotti RJ, Simons-Morton B (2013). Prevalence and patterns of polysubstance use in a nationally representative sample of 10th graders in the United States. J Adolesc Health.

[4] Schulenberg JE, Johnston LD, O'Malley PM, Bachman JG, Miech RJ, Patrick ME (2018). Monitoring the Future National Survey Results on Drug Use-2014: Volume II, College Students and Adults Ages.

[5] Winters KC, Lee CS (2008). Likelihood of developing an alcohol and cannabis use disorder during youth: association with recent use and age. Drug Alcohol Depend.

[6] Cooley-Strickland M, Quille TJ, Griffin RS, Stuart EA, Bradshaw CP, Furr-Holden D (2009). Community violence and youth: affect, behavior, substance use, and academics. Clin Child Fam Psychol Rev.

[7] Green KM, Musci RJ, Johnson RM, Matson PA, Reboussin BA, Ialongo NS (2016). Outcomes associated with adolescent marijuana and alcohol use among urban young adults: a prospective study. Addict Behav.

[8] Lardier DT (2019). Substance use among urban youth of color: exploring the role of community-based predictors, ethnic identity, and intrapersonal psychological empowerment. Cultur Divers Ethnic Minor Psychol.

[9] Amaro H, Raj A, Vega RR, Mangione TW, Perez LN (2001). Racial/Ethnic disparities in the HIV and substance abuse epidemics: communities responding to the need. Public Health Reports.

[10] Johnson EI, Copp JE, Bolland AC, Bolland JM (2020). Substance use profiles among urban adolescents: the role of family-based adversities. J Child Fam Stud.

[11] Gfroerer J, Wright D, Kopstein A (1997). Prevalence of youth substance use: the impact of methodological differences between two national surveys. Drug Alcohol Depend.

[12] Dembo R, Briones-Robinson R, Barrett K, Winters KC, Schmeidler J, Ungaro RA, Karas L, Belenko S, Gulledge L (2013). The mental health, substance use, and delinquency among truant youths in a brief intervention project: a longitudinal study. J Emot Behav Disord.

[13] Johnson TP (2014). Sources of error in substance use prevalence surveys. Int Sch Res Notices.

[14] Swaim RC, Beauvais F, Chavez EL, Oetting ER (1997). The effect of school dropout rates on estimates of adolescent substance use among three racial/ethnic groups. Am J Public Health.

[15] Weisner C, Schmidt L, Tam T (1995). Assessing bias in community-based prevalence estimates: towards an unduplicated count of problem drinkers and drug users. Addiction.

[16] Hallfors D, Vevea J, Iritani B, Cho H, Khatapoush S, Saxe L (2002). Truancy, grade point average, and sexual activity: a meta-analysis of risk indicators for youth substance use. J Sch Health.

[17] Gakh M, Coughenour C, Assoumou BO, Vanderstelt M (2020). The relationship between school absenteeism and substance use: an integrative literature review. Subst Use Misuse.

[18] Bronfenbrenner U, Vasta R (1992). Ecological systems theory. Six Theories of Child Development: Revised Formulations and Current Issues.

[19] Cicchetti D, Lynch M (1993). Toward an ecological/transactional model of community violence and child maltreatment: consequences for children's development. Psychiatry.

[20] Bernard DL, Calhoun CD, Banks DE, Halliday CA, Hughes-Halbert C, Danielson CK (2020). Making the “C-ACE” for a culturally-informed adverse childhood experiences framework to understand the pervasive mental health impact of racism on black youth. Journ Child Adol Trauma.

[21] Eisman AB, Stoddard SA, Heinze J, Caldwell CH, Zimmerman MA (2015). Depressive symptoms, social support, and violence exposure among urban youth: a longitudinal study of resilience. Dev Psychol.

[22] Floyd LJ, Brown Q (2013). Attitudes toward and sexual partnerships with drug dealers among young adult African American females in socially disorganized communities. J Drug Issues.

[23] Yabiku S, Kulis S, Marsiglia FF, Lewin B, Nieri T, Hussaini S (2007). Neighborhood effects on the efficacy of a program to prevent youth alcohol use. Subst Use Misuse.

[24] Kubrin C, Wo J, Piquero AR (2015). Social disorganization theory's greatest challenge: linking structural characteristics to crime in socially disorganized communities. The Handbook of Criminological Theory.

[25] Shaw CR, McKay HD (1970). Juvenile delinquency and urban areas. Am Sociol Rev.

[26] Kotlaja MM, Wright EM, Fagan AA (2018). Neighborhood parks and playgrounds: risky or protective contexts for youth substance use?. J Drug Issues.

[27] Lardier Jr DT, MacDonnell M, Barrios VR, Garcia-Reid P, Reid RJ (2018). The moderating effect of neighborhood sense of community on predictors of substance use among Hispanic urban youth. J Ethn Subst Abuse.

[28] Rice HM, Musil C, Kretschmar J, Warner C (2018). Neighborhood disorganization, social support, substance use and functioning amongst adolescents; an analysis of the Ohio Behavioral Health Juvenile Justice initiative. J Adolesc Health.

[29] Breland-Noble AM, Bell CC, Burriss A, Poole HK, The AAKOMA Project Adult Advisory Board (2012). J Child Fam Stud.

[30] Romley JA, Cohen D, Ringel J, Sturm R (2007). Alcohol and environmental justice: the density of liquor stores and bars in urban neighborhoods in the United States. J Stud Alcohol Drugs.

[31] Fagan AA, Wright EM, Pinchevsky GM (2015). Exposure to violence, substance use, and neighborhood context. Soc Sci Res.

[32] Martin SL, Sigda KB, Kupersmidt JB (2016). Family and neighborhood violence: predictors of depressive symptomatology among incarcerated youth. Prison J.

[33] Butcher F, Galanek JD, Kretschmar JM, Flannery DJ (2015). The impact of neighborhood disorganization on neighborhood exposure to violence, trauma symptoms, and social relationships among at-risk youth. Soc Sci Med.

[34] Sun S, Crooks N, DiClemente RJ, Sales JM (2020). Perceived neighborhood violence and crime, emotion regulation, and PTSD symptoms among justice-involved, urban African-American adolescent girls. Psychol Trauma.

[35] Slopen N, Fitzmaurice GM, Williams DR, Gilman SE (2012). Common patterns of violence experiences and depression and anxiety among adolescents. Soc Psychiatry Psychiatr Epidemiol.

[36] Erskine HE, Moffitt TE, Copeland WE, Costello EJ, Ferrari AJ, Patton G, Degenhardt L, Vos T, Whiteford HA, Scott JG (2014). A heavy burden on young minds: the global burden of mental and substance use disorders in children and youth. Psychol Med.

[37] (2016). Paterson City, New Jersey. United States Census Bureau.

[38] (2018). HIV/AIDS cases in statewide population groups. State of New Jersey, Department of Health.

[39] (2016). A research report: the profile of substance use and other indicators of well-being among youth in Paterson, NJ. P-CASA.

[40] Division of Alcoholic Beverage Control. State of New Jersey, Department of Law & Public Safety.

[41] Castillo-Carniglia A, Pear V, Tracy M, Keyes K, Cerdá M (2019). Limiting alcohol outlet density to prevent alcohol use and violence: estimating policy interventions through agent-based modeling. Am J Epidemiol.

[42] Rowland B, Toumbourou JW, Livingston M (2015). The association of alcohol outlet density with illegal underage adolescent purchasing of alcohol. J Adolesc Health.

[43] Rowland B, Evans-Whipp T, Hemphill S, Leung R, Livingston M, Toumbourou J (2016). The density of alcohol outlets and adolescent alcohol consumption: an Australian longitudinal analysis. Health Place.

[44] Davis T (2018). 30 NJ towns with the most heroin abuse in new 2018 report. Patch Labs.

[45] Earnshaw VA, Elliott MN, Reisner SL, Mrug S, Windle M, Emery ST, Peskin MF, Schuster MA (2017). Peer victimization, depressive symptoms, and substance use: a longitudinal analysis. Pediatrics.

[46] Jackson JM, Seth P, DiClemente RJ, Lin A (2015). Association of depressive symptoms and substance use with risky sexual behavior and sexually transmitted infections among African American female adolescents seeking sexual health care. Am J Public Health.

[47] Eysenbach G (2004). Improving the quality of web surveys: the Checklist for Reporting Results of Internet E-Surveys (CHERRIES). J Med Internet Res.

[48] Eysenbach G (2012). Correction: improving the quality of web surveys: the Checklist for Reporting Results of Internet E-Surveys (CHERRIES). J Med Internet Res.

[49] Valaitis RK (2005). Computers and the internet: tools for youth empowerment. J Med Internet Res.

[50] Curry L, Nunez-Smith M (2014). Mixed Methods in Health Sciences Research: A Practical Primer (Mixed Methods Research Series).

[51] Muhib FB, Lin LS, Stueve A, Miller RL, Ford WL, Johnson WD, Smith PJ (2016). A venue-based method for sampling hard-to-reach populations. Public Health Rep.

[52] Verdery A, Weir S, Reynolds Z, Mulholland G, Edwards J (2019). Estimating hidden population sizes with venue-based sampling: extensions of the generalized network scale-up estimator. Epidemiology.

[53] Etikan I (2016). Comparison of convenience sampling and purposive sampling. Am J Theor Appl Stat.

[54] Tongco MD (2007). Purposive sampling as a tool for informant selection. Ethnobot Res App.

[55] Biernacki P, Waldorf D (2016). Snowball sampling: problems and techniques of chain referral sampling. Sociol Methods Res.

[56] Brawner BM, Volpe EM, Stewart JM, Gomes MM (2013). Attitudes and beliefs toward biobehavioural research participation: voices and concerns of urban adolescent females receiving outpatient mental health treatment. Ann Hum Biol.

[57] Mustanski B, Coventry R, Macapagal K, Arbeit MR, Fisher CB (2017). Sexual and gender minority adolescents' views on HIV research participation and parental permission: a mixed-methods study. Perspect Sex Reprod Health.

[58] Brawner BM, Sutton MY (2017). Sexual health research among youth representing minority populations: to waive or not to waive parental consent. Ethics Behav.

[59] Liu C, Cox RB, Washburn IJ, Croff JM, Crethar HC (2017). The effects of requiring parental consent for research on adolescents' risk behaviors: a meta-analysis. J Adolesc Health.

[60] Kevern J, Webb C (2001). Focus groups as a tool for critical social research in nurse education. Nurse Educ Today.

[61] Patton QM (2001). Qualitative Research & Evaluation Methods. 3rd Ed.

[62] Davis MS (2019). Youth Risk Behavior Survey (YRBS). The Concise Dictionary of Crime and Justice.

[63] Derogatis LR (1982). Brief Symptom Inventory (BSI). APA PsycTests.

[64] Handley ED, Rogosch FA, Guild DJ, Cicchetti D (2015). Neighborhood disadvantage and adolescent substance use disorder: the moderating role of maltreatment. Child Maltreat.

[65] Esteban-Cornejo I, Carlson JA, Conway TL, Cain KL, Saelens BE, Frank LD, Glanz K, Roman CG, Sallis JF (2016). Parental and adolescent perceptions of neighborhood safety related to adolescents' physical activity in their neighborhood. Res Q Exerc Sport.

[66] Clark R, Coleman AP, Novak JD (2004). Brief report: initial psychometric properties of the everyday discrimination scale in black adolescents. J Adolesc.

[67] Anderson JC, Funk JB, Elliott R, Smith PH (2003). Parental support and pressure and children's extracurricular activities: relationships with amount of involvement and affective experience of participation. J Appl Dev Psychol.

[68] Graham JW (2009). Missing data analysis: making it work in the real world. Annu Rev Psychol.

[69] Hoyle RH (1995). Structural Equation Modeling: Concepts, Issues, and Applications.

[70] Hill MC, Tiedeman CR (2005). Evaluating model fit. Effective Groundwater Model Calibration: With Analysis of Data, Sensitivities, Predictions, and Uncertainty.

[71] Castleberry A (2014). NVivo 10 [software program]. Version 10. QSR International; 2012. Am J Pharm Educ.

[72] (2016). SAS user's guide: statistics. SAS Institute Inc.

[73] Ott MA, Campbell J, Imburgia TM, Yang Z, Tu W, Auerswald CL (2018). Community engagement and venue-based sampling in adolescent male sexually transmitted infection prevention research. J Adolesc Health.

[74] Wingood G, DiClemente R (2008). The ADAPT-ITT model: a novel method of adapting evidence-based HIV interventions. J Acquir Immune Defic Syndr.

[75] Murray LF, Belenko S (2005). CASASTART: a community-based, school-centered intervention for high-risk youth. Subst Use Misuse.

[76] Sussman S, Dent CW, Stacy AW (2002). Project towards no drug abuse: a review of the findings and future directions. Am J Health Behav.

[77] Reese LE, Vera EM (2016). Culturally relevant prevention. Couns Psychol.

[78] (2003). Preventing drug abuse among children and adolescents: a research-based guide for parents, educators, and community leaders. 2nd edition. National Institute on Drug Abuse.

